# Color behavior of composite resin enhanced with different shapes of new antimicrobial polymer coated nanoparticles

**DOI:** 10.1186/s12903-023-03495-w

**Published:** 2023-10-19

**Authors:** Ghada Naguib, Hisham Mously, Walaa Magdy, Abdulelah Binmahfooz, Osama Qutub, Maher Hajjaj, Mohamed Tharwat Hamed

**Affiliations:** 1 Department of Restorative Dentistry, Faculty of Dentistry, King Abdulaziz University, Jeddah, Saudi Arabia; 2 Department of Oral Biology, Cairo University School of Dentistry, Cairo, Egypt; 3 Department of Oral and Maxillofacial Prosthodontics, Faculty of Dentistry, King Abdulaziz University, Jeddah, Saudi Arabia; 4 Department of Oral and Maxillofacial Prosthodontics, Faculty of Dentistry, King Abdulaziz University, P.O Box 80209, 21589 Jeddah, Saudi Arabia; 5 Department of Fixed Prosthodontics, Cairo University School of Dentistry, Cairo, Egypt

**Keywords:** Magnesium oxide, Nano-filled composite, Nanowires composite, Color stability, Staining, Spectrophotometer

## Abstract

**Background:**

Zein-coated magnesium oxide nanoparticles (zMgO NPs) demonstrate a potent antimicrobial effect, endorsing it as a compelling additive to dental materials formulations for oral health care advances. However, currently there is no data on the imprint of zMgO NPs on the color permanence of dental composites. The objective of this study is to evaluate the color stability of different types of composite enhanced with antimicrobial zein-coated magnesium oxide nanoparticles (zMgO NPs) of different shapes before and after thermocycling.

**Methods:**

Two hundred composite samples were divided into four groups: Gp1: Tetric N-Flow with zMgO nanowires, Gp2: Tetric N-Flow with zMgO nanospheres, Gp3: Tetric N-Ceram with zMgO nanowires; Gp4: Tetric N-Ceram with zMgO nanospheres. Each group was subdivided into 5 subgroups (*n* = 10) with concentrations of zMgO NPs 0%, 0.3%, 0.5%, 1% and 2%. The characterization of the modified composite containing the zMgO was done via X-ray Diffraction, Field Emission Scanning Electron Microscopy (FESEM), and Fourier Transform Infrared Spectroscopy (FTIR). Colorimetric evaluation was performed through spectrophotometry with a white background. Samples underwent color assessment using a spectrophotometer, followed by thermocycling, and then another color assessment.

**Results:**

FESEM analysis showed a uniform distribution of the zMgO nanoparticles in the composite and FTIR illustrated no change in the spectra. However, the XRD spectra exhibited an amorphous pattern in the composite enhanced with zMgO NPs. There was no compelling discrepancy in color variation ΔE among the different groups before and after thermocycling (*p* > 0.05). A statistically notable variation in ΔL was found amid the control and N-Flow and N-Ceram with 2% zMgO nanospheres before and after thermocycling respectively (*p* < 0.05). While after thermocycling, there was a statistically significant difference in Δa in N-Flow and N-Ceram wires amid the control and the different groups (*p* < 0.05). Additionally, after thermocycling there was a statistically significant difference in Δb in N-Flow and N-Ceram wires between the control and the different groups (*p* < 0.05). The Tukey test exhibited no variation among the groups with different zMgO concentrations (*p* > 0.05).

**Conclusion:**

Enhancing N-Flow and N-Ceram composite with antimicrobial zMgO nanowires and nanospheres did not alter the total color stability of the materials before and after thermocycling.

## Introduction

With the increasing emphasis on esthetics in the recent decades, the popularity of dental esthetic restorative materials has risen among dentists and patients [1, 2]. Esthetic restorative materials are intended to mimic the appearance of the natural tooth, and their discoloration or staining represents a significant challenge. The esthetic appearance of a restorative material is directly related to its color match and color stability; a proper color match with the adjacent tooth is essential over a long term [3]. This has resulted in the need for esthetic restorative materials with superior characteristics to withstand the harsh environmental oral changes [2].

Currently, dental resin composites play an important role in restorative dentistry due to the advantages of their properties [4]. These materials can replicate the beauty of the natural tooth using conservative methods [5], which has contributed to their increased use in anterior and posterior tooth restorations [1–5]. However, evidence on the discoloration potential of composite resin restorations is mostly limited to that furnished by manufacturers, with a lack of scientific data.

Some authors have studied the discoloration of newly developed resin composite materials [3–10]. Color transformation and surface degradation detected clinically are related to both the nature of the composite restoration and the patients’ habits. Absorption of moisture by composites due to the nature of the resin matrix can make composite restorations vulnerable to penetration by various staining agents [6, 11].

Staining of composite restorations can be attributed to three types of discolorations: external discoloration due to plaque buildup and superficial stains (extrinsic stain), surface or subsurface color change with minor infiltration of staining agents (absorption) and intrinsic discoloration due to physicochemical reactions in the restoration bulk [12].

The extrinsic and intrinsic factors include intake of beverages, medications, dentifrices, and tooth bleaching products [7, 10], and their effect increases over time [11]. It was reported that bleaching mouthwash could greatly affect the color stability and surface microhardness of the nanohybrid resin composite [13]. Staining sensitivity is associated with the type and arrangement of the resin matrix together with the characteristics and dimensions of the filler particles [14]. Furthermore, resin-based restorations have been reported to have a higher resistance to color change [7], and nano-filled composites have shown favorable outcomes [8].

Nanotechnology is the science at a nanoscale (one-billionth of a meter) [15]. A material smaller than 100 nm in one dimension is defined as a nanomaterial [16, 17]. The most characteristic feature of nanomaterials is their large surface to volume ratio. They also have a very potent antimicrobial action against bacterial biofilm [17, 18], and can fill the gaps between the inter-polymeric chains, resulting in increased mechanical and physical strength [19].

Nanomaterials can be synthesized in a variety of ways, depending on a variety of factors such as the dimensions of the materials being created. Researchers divided nanoparticles into: zero-dimensional, one-dimensional, two-dimensional, and three-dimensional [20]. Zero-dimensional nanoparticles are defined as the nanostructure that has all dimensions in the nano-range. New and enhanced structural and physico-chemical properties were generated by the production of one-dimensional nanomaterials, e.g. nanowires. Nanowires enhance the properties of dental composites and are used to treat carious lesions and repair enamel structure [19]. Similarly, nanospheres simulate nanostructures in tooth development, furnish the base for formation and growth of enamel crystals, and yield growth factors with prolonged curative power [21].

Since nano-dentistry focuses on improving oral health by introducing novel restorative nanomaterials for treatment of caries [17], recently nanoparticles have been used as fillers in dental composite resin to improve its physical properties, especially its light spectrum management [16, 22].

Magnesium (Mg) has long since been identified as a vital element for the body’s health. Meanwhile, the new characteristics of nanosized MgO entitled them to be the fundamental element for superlative human health [23]. Researchers stated that the antimicrobial activity of MgO nanoparticles against Gram-positive and Gram-negative microorganisms has a direct relationship to their concentration and sizes [24–27].

However, a common problem with nanoparticles is their tendency to agglomerate to decrease their surface energy, which in turn affects their antimicrobial and optical properties [15, 28]. For this reason, good dispersion of NPs in the matrix is considered the main key for an effective nanomaterial. Zein is a natural corn polymer that has many distinctive properties that recommend it to be utilized in drug coating industry. It can be used in the nano form to coat the MgO nanoparticles in order to prevent their aggregation [29].

Lately, magnesium oxide nanoparticles (MgO NPs) have shown a potent antimicrobial effect when added to different dental materials [30–32]. Antimicrobial zein-coated MgO nanoparticles (zMgO NPs) have been proven to be safe with regards to biochemistry and cytotoxicity, endorsing it as a compelling additive to new dental materials formulations for oral health care advances [33].

Nevertheless, there is limited data on the effect of adding zein-coated magnesium oxide nanoparticles (zMgO NPs) on the properties of restorative materials. In addition, there is no data on the imprint of zMgO NPs on the color permanence of dental composites. The purpose of this study is to assess and analyze the color stability of different dental composite resins enhanced with zMgO NPs of different shapes and concentrations before and after thermocycling. The null hypothesis is that adding different shapes and concentrations of zMgO NPs to different types of resin composite does not change the color stability of these composite esthetic materials. However, the alternate hypothesis states that significant changes occur in the color stability of resin composite materials reinforced with zMgO NPs.

## Materials and methods

### Ethical approval

An ethical approval was obtained from the Research Ethics Committee at King Abdulaziz University, Faculty of Dentistry (#100–10-17).

### Study design

**Fig. 1:**
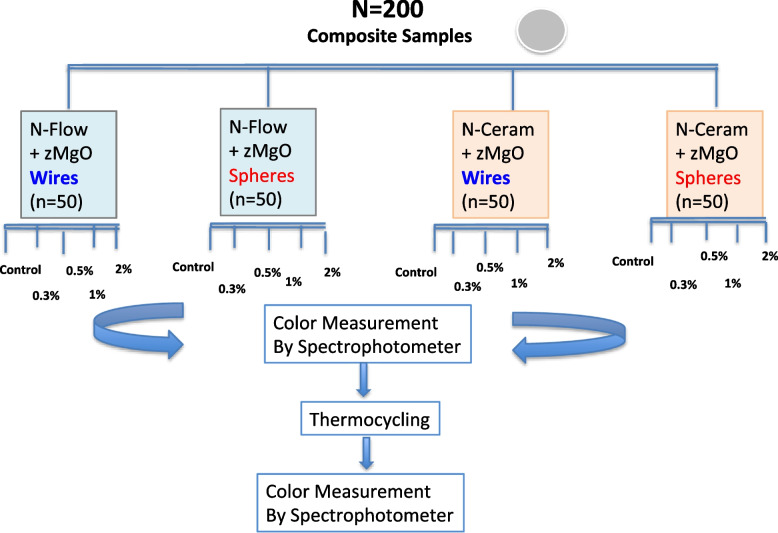
Diagrammatic flowchart of the study groups depending on composite materials and zMgO shape and concentrations

In this study, 200 composite samples were divided into four main groups: Group 1: Tetric N-Flow reinforced with zein-coated magnesium oxide nanoparticles (zMgO NPs) nanowires, Group 2: Tetric N-Flow reinforced with (zMgO NPs) nanospheres, Group 3: Tetric N-Ceram reinforced with (zMgO NPs) nanowires; Group 4: Tetric N-Ceram reinforced with (zMgO NPs) nanospheres. Each group was subdivided into 5 subgroups (*n* = 10) with different concentrations of zMgO NPs at 0%, 0.3%, 0.5%, 1% and 2% (Fig. 1). These groups would then be subjected to a color assessment, thermocycled, and then another color assessment.

### Materials

The following two composite materials were evaluated: Tetric N-Flow and Tetric N-Ceram (Ivoclar Vivadent). Magnesium oxide nanoparticles wires and spheres were synthesized and coated with zein polymer as described earlier (Table 1) [34].

**Table 1:** Manufacturer and composition of materials used in this study

Material (Manufacturer)	Composition
Tetric N-Ceram, Ivoclar Vivadent AG, Schaan, Liechtenstei	Dimethacrylates (19–20 wt. %), Fillers contain barium glass, ytterbium trifluoride, mixed oxide and copolymers (80–81 wt. %)
Tetric N-Flow, Ivoclar Vivadent AG, Schaan, Liechtenstein	UDMA, bis-GMA, TEGDMA
zMgO Nanoparticles (Lab prepared)	MgO Nanoparticles (Nanowires and Nanospheres) coated with zein nanopolymer

In this study, a conventional flowable composite (Tetric-N flow, Ivoclar/Vivadent, Liechtenstein), two-step self-etch adhesive (Clearfil SE bond 2, Kuraray Noritake Dental, Japan) and MgO nanowires (particle size 40nm diameter and 100nm length) synthesized by microwave in the two different concentrations were used [30]. The MgO nanofillers were weighed using a balance accurate to 0.0001g (BEL Engineering, Monza, Italy) and were added to the flowable composite in the ratio of 0.3% and 0.5% by weight.

### Coating of MgO nanoparticles

MgO nanowires (particle size 40nm diameter and 100nm length) manufactured by microwave in the two different concentrations were used [30]. As reported in a preceding study by Naguib et al. [31], pH-controlled nanoprecipitation was applied for zein-coating of MgO nanoparticles. Cogent reagents were received from Sigma-Aldrich, St. Louis, MO, USA. Zein polymer (0.02g) was mixed in a solution of ethanol and 0.1 NaOH (93.7% (v/v)). By applying droplet infusion, zein droplets were interpolated with 15 ml blend of MgO (0.02g) and polyvinyl alcohol (PVA) at 0.9% (w/v) under 750W ultrasonic shear, 20 kHz frequency and 10°C temperature. The blend was mixed magnetically at a rate of 500rpm. Afterwards, the blend was centrifuged twice at 3,000rpm for 45 min in order to attain pure nanoparticles as well as to eliminate excess polyvinyl alcohol. Subsequently, the supernatant was discarded and a 5ml buffer was used to dissolve the formed pellet. Later, the mixture was lyophilized (VirTis Bench Top Lyophilizer, SP Industries, Stone Ridge, NY, USA) [34, 35] after the addition of 2% (w/v) of trehalose. Then, the MgO nanoparticles (4:1 by weight) were added to a blend of zein with polyvinyl alcohol (2:1 by weight). As described previously by Naguib et al. [31], the two mixtures of MgO zein and polyvinyl alcohol were placed on the magnetic stirrer and stirred for 30 min. The polyvinyl alcohol was volatilized, after which the mix was then centrifuged and freeze-dried.

### Characterization of the dental composite modified with zein-modified MgO nanoparticles

#### X-Ray diffraction analysis

To study the type of phases and crystalline structure of zMgO NPs, an X-Ray diffractometer (XRD; Rigaku, Ultima IV, Japan) with Cu-Kα X-ray radiation (λ = 1.542 A^◦^) was used. The crystalline phase and patterns of both the N-Flow composite after integration of the zMgO NPs and the zMgO NPs alone underwent X-Ray Diffraction analysis. The XRD spectra were scanned in the 2θ range of 10–80°.

#### Field emission scanning electron microscopy

The surface morphology and the distribution of zMgO nanoparticles in the N-Flow composite specimens were investigated by using Field Emission Scanning Electron Microscopy (FESEM) (JEOL JSM-7600F, JEOL Ltd. Tokyo, Japan), under an ultra-high vacuum (~ 10^−6^ mbar).

#### Fourier transform infrared spectroscopy

The FTIR spectra of the composite before and after integration of the zMgO NPs were evaluated. FTIR Type 8000 Series Fourier Transformation (Shimadzu Co., Japan) was used to record the Fourier transformation of the infrared spectra by utilizing KBr plates in the absorbance mode at a wavenumber range of 500–4000 cm^−1^ under identical conditions.

### Specimen preparation

Composite material was mixed with the different concentrations of zMgO NPs (0.0%, 0.3%, 0.5%,1% and 2% by weight). Specimens were prepared as a standardized disc of 10 mm diameter and 2 mm thickness by filling the restorative material in a custom Teflon mold and gently pressing it with glass slides above and below the mould to ensure a smooth surface. The composite resins were light-cured from a zero-distance using an LED curing device (3M ESPE Elipar ™) with a 10-mm internal diameter, 430–480 nm wavelength and 1470 mW/cm2 irradiance, in congruence with the manufacturer’s technique. The specimens were allocated randomly to the sub-groups, such that each subgroup contained 10 discs (*n* = 10) (Fig. 1) [36].

### Thermocycling

To simulate an old (aged) restoration, all restorations were thermocycled for approximately 10,000 cycles in baths containing distilled water at 5–55°C, with a 32-s dwell time in each bath and a 14-s interval between baths. Thermal stress was applied concurrently using a thermocycler machine (JULABO GmbH, Seelbach, Germany).

### Color assessment

Color differences were classified and calculated according to the Commission Internationale de l’Eclairage L*, a*, b* (CIE-Lab) colorimetric system. The color stability of the specimens was measured twice: before and after thermocycling. The tests were performed using a color spectrophotometer (X rite Color Eye 7000a /Net Profiler Ready/ USA) to obtain the Δ*E* value. The equipment was adjusted to compute three color parameters: L* represents a range of lightness (0 black to 100 white), while chroma is expressed as a* and b* exhibiting red for a positive a*, green for a negative a*, yellow for a positive b*, and blue for a negative b*.

The following equation was applied using three points from each sample (L, lightness; a, red/green axis; and b, yellow/blue axis), and the difference between the two readings, or the delta (Δ) value, was calculated at each point.

$${\varvec{\Delta}}\mathbf{E}(\mathbf{L}\mathbf{*}\mathbf{a}\mathbf{*}\mathbf{b})=\sqrt{({\varvec{\Delta}}\mathbf{L})^2+({\varvec{\Delta}}\mathbf{a})^2+({\varvec{\Delta}}\mathbf{b})^2}$$

During assessments, external lights were turned off and, after an adaptation period, the observers scored the color-matching between the universal shade and shaded composite discs which were in edge contact [37].

### Statistical analysis

Data was analyzed by adopting a statistical software package (SPSS Statistics Version 26.0, IBM, New York, NY, USA). The color change (∆E) values were determined by Two-way ANOVA followed by Tukey's test. Significant variations were recognized amid the composite groups at *p* < 0.05.

Regression analysis was performed for intra-group comparisons of color coordinates and to evaluate changes in color and color parameters (ΔE*, color difference; ΔL*, change in lightness; Δa* and Δb* change in chroma) after thermocycling. Pearson’s correlation coefficient was computed (*p* < 0.05). The confidence level was set as 95%.

## Results

### Characterization

#### X-Ray diffraction analysis

**Fig. 2:**
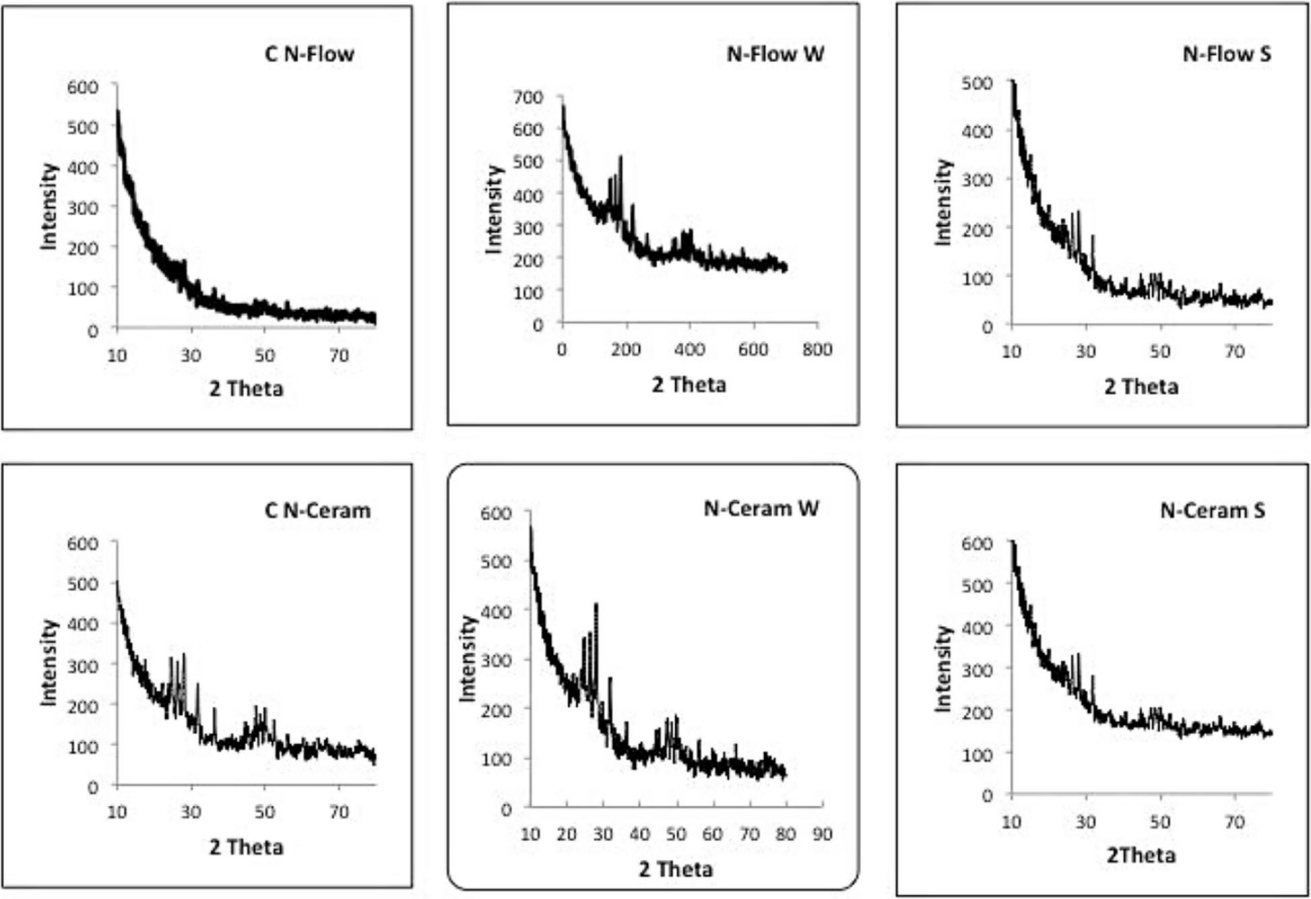
X-Ray Diffraction profiles for the nanocomposites used in the study. C N-Flow: Control N-Flow; C N-Ceram: Control N-Ceram; N-Flow W: N-Flow Wires; N-Ceram W: N-Ceram Wires; N-Flow S: N-Flow Spheres; N-Ceram S: N-Ceram Spheres

Figure 2 depicts the XRD spectrum of the N-Flow and N-Ceram composite resin with zMgO nanoparticles wires and spheres compared to the control. Diffraction peaks appear to be obscured by the amorphous nature of the composite polymer, which could be due to the small percentage of the zMgO NPs.

#### Field emission scanning electron microscopy

**Fig. 3:**
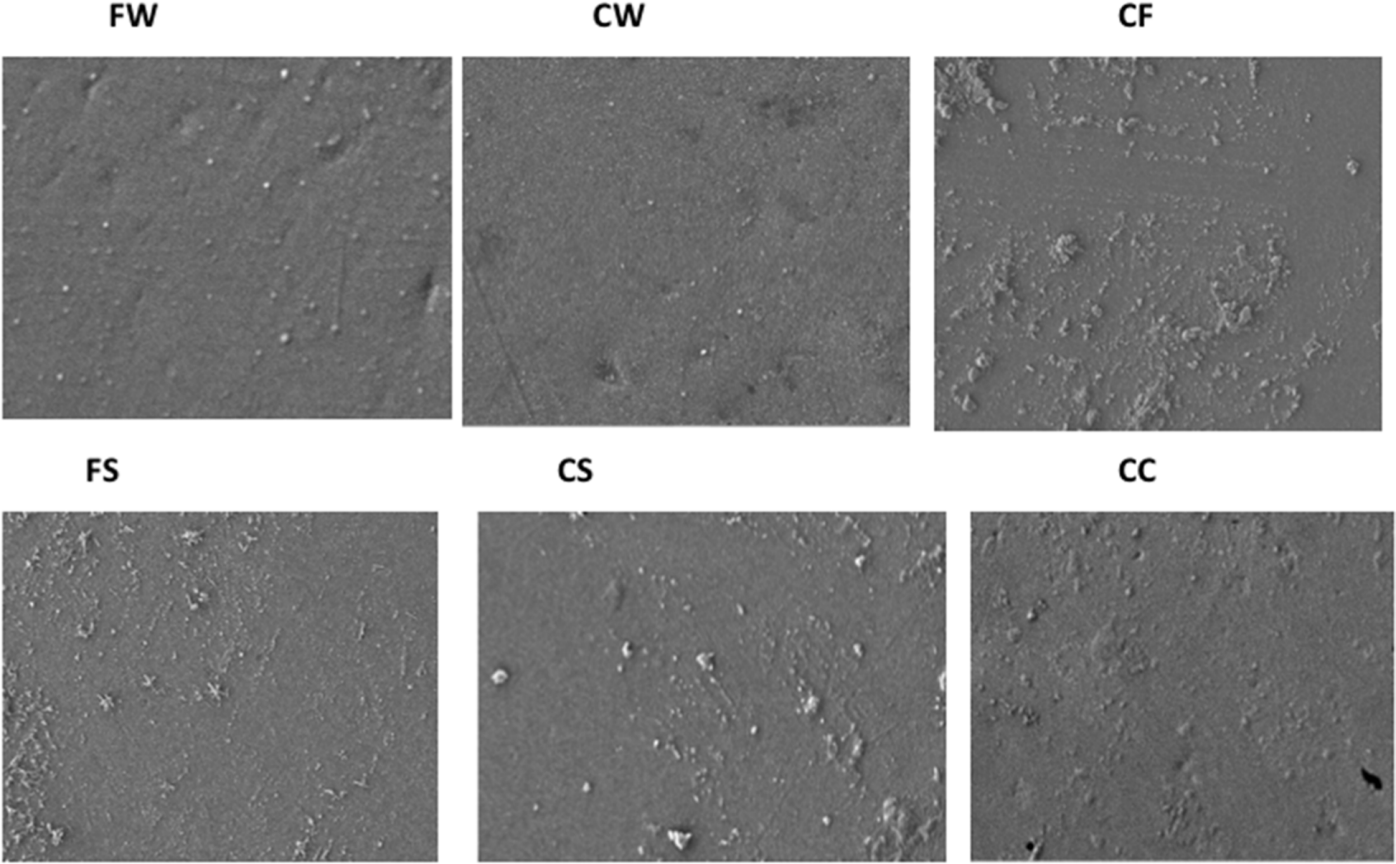
Field Emission Scanning Electron Micrographs for the nanocomposites used in the study: FW: N-Flow with zMgO NPs wires, CW: N-Ceram with zMgO NPs wires, FS: N-Flow with zMgO NPs spheres, CS: N-Ceram with zMgO NPs spheres, CF: N-Flow Control, CC: Control N-Ceram

Figure 3 presents the morphological characteristics of N-Flow composite before and after adding zMgO NPs. Micrographs show that the zMgO nanoparticles are uniformly distributed along the entire surface of the N-Flow composite specimen. However, due to the non-conducting disposition of the composite resin material, very high magnification images could not be obtained.

#### Fourier Transform Infrared Spectroscopy (FTIR)

**Fig. 4:**
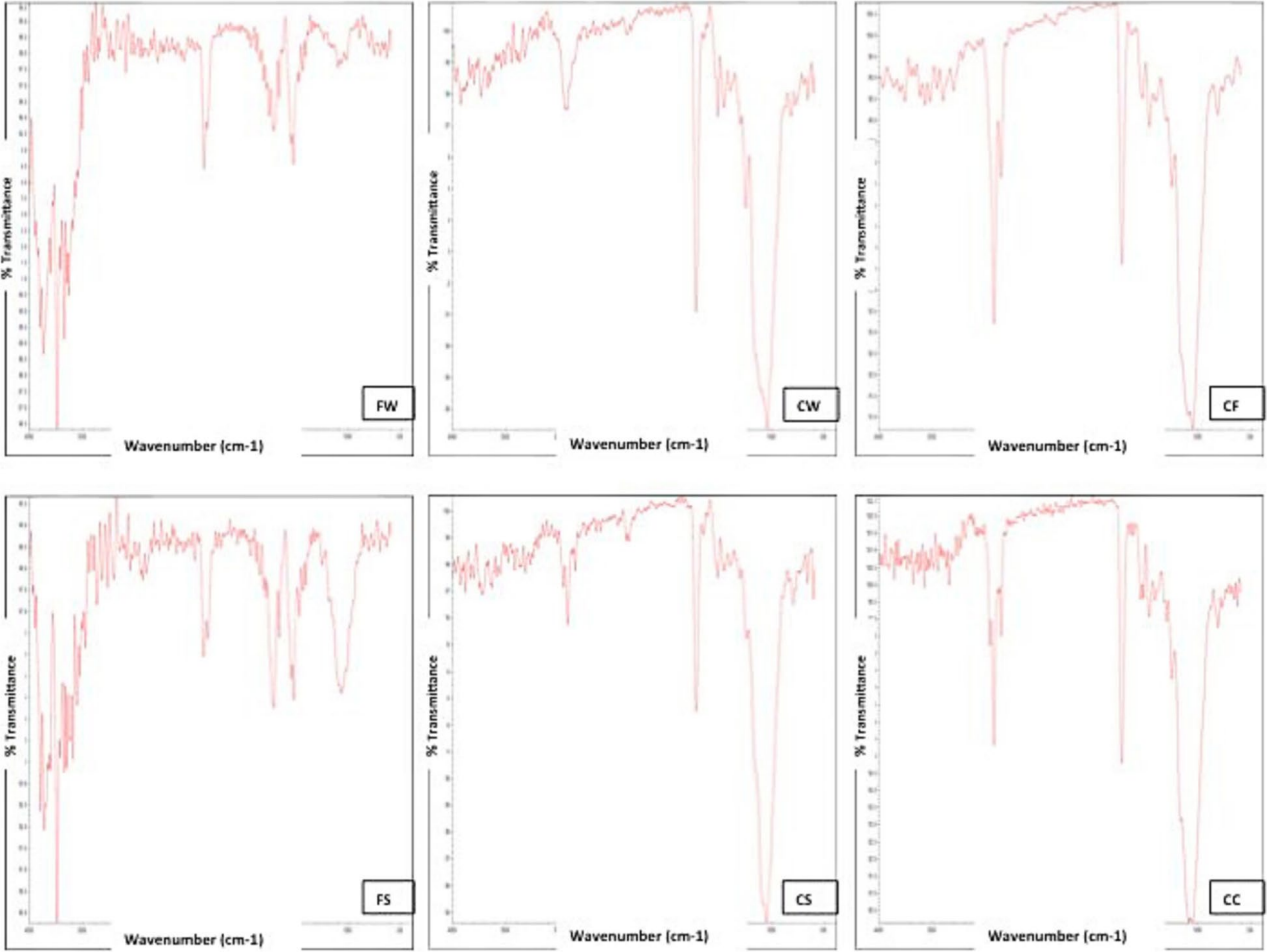
Fourier Transform Infrared Spectroscopy (FTIR) spectra for the nanocomposites used in the study: FW: N-Flow with zMgO NPs wires, CW: N-Ceram with zMgO NPs wires, FS: N-Flow with zMgO NPs spheres, CS: N-Ceram with zMgO NPs spheres, CF: N-Flow Control, CC: Control N- Ceram

The FTIR spectra of N-Flow with zMgO NPs and the N-Flow alone (control) nearly mirror each other. There is a stretching at 1000 cm-^1^ corresponding to C-O band of silicate as a result of the presence of the inorganic filler particles. Additionally, the appearance of a slight stretching was found at 1700 cm-^1^ band corresponding to the C = O group of esters, with a shortening of the band at 2800 cm-^1^ corresponding to C-H group. This indicates that the zMgO NPs does not alter the structure of N-Flow composite (Fig. 4).

### ΔE* Values

**Fig. 5:**
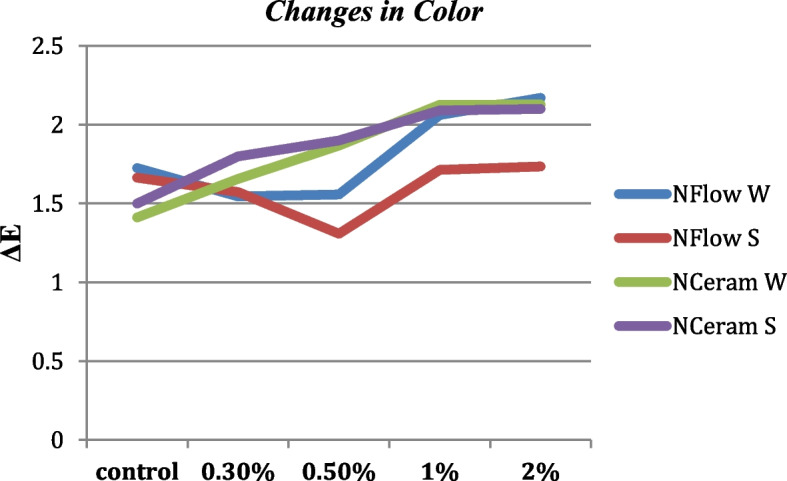
Changes in color (ΔE) of composites reinforced with zMgO NPs after thermocycling. NFlow W: Tetric-N-Flow wires nanoparticles; NFlow S: Tetric-N-Flow spherical nanoparticles; NCeram W: Tetric-N-Ceram wires nanoparticles; NCeram S: Tetric-N-Ceram spherical nanoparticles

Results showed no significant variation in color ΔE amid the different groups of composites before and after thermocycling (p > 0.05). Pearson coefficient showed a weak correlation of ΔE in groups 1, 2, 3, and 4 respectively (r = 0.19, 0.19, 0.13 and 0.14) (Fig. 5).

### ΔL* (Brightness) Values

**Fig. 6:**
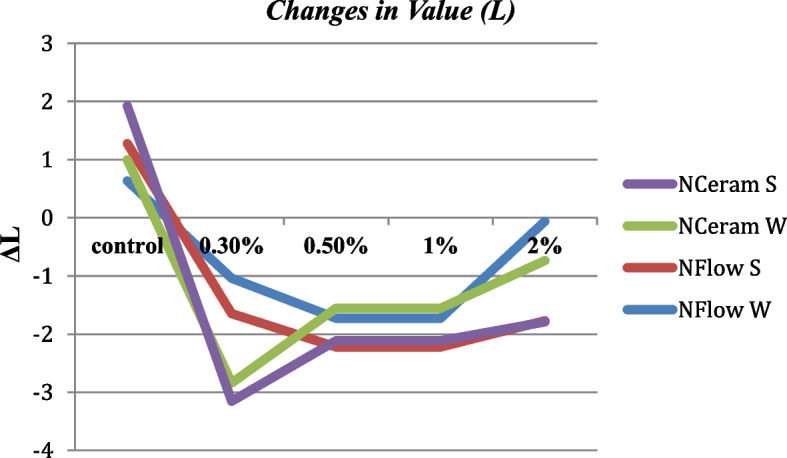
Changes in value (ΔL) of MgO nanoparticle composites after thermocycling. NFlow W: Tetric-N-Flow wires nanoparticles; NFlow S: Tetric-N-Flow spherical nanoparticles; NCeram W: Tetric-N-Ceram wires nanoparticles; NCeram S: Tetric-N-Ceram spherical nanoparticles

**Fig. 7:**
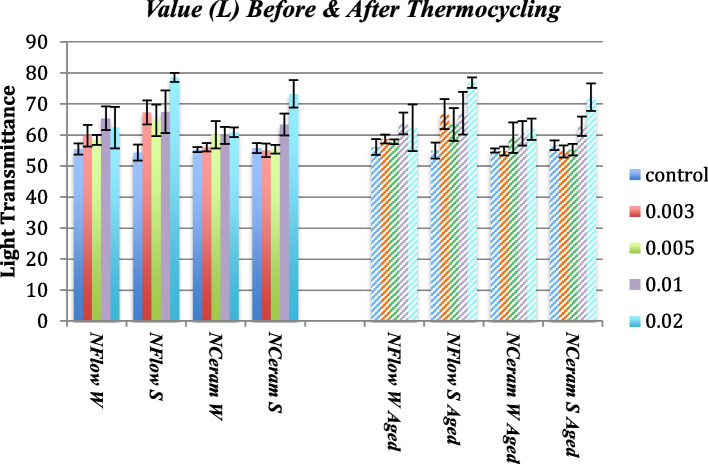
Changes in value (L) of MgO nanoparticle composites before and after thermocycling. NFlow W: Tetric-N-Flow wires nanoparticles; NFlow S: Tetric-N-Flow spherical nanoparticles; NCeram W: Tetric-N-Ceram wires nanoparticles; NCeram S: Tetric-N-Ceram spherical nanoparticles

There was a significant variation amid the different composite groups (*p* = 0.07). Tukey test indicated a statistically significant variation amid the control and N-Flow with 2% zMgO spheres and N- Ceram with 2% zMgO spheres before and after thermocycling respectively (*p* = 0.02, *p* = 0.01, *p* = 0.02, *p* = 0.01) (Figs. 6 and 7). ΔL* values were in the range of -0.3 to 1.2. Pearson coefficient showed a weak correlation of ΔL in groups 1, 2, 3, and 4, respectively (r = 0.1, 0.3, 0.3 and 0.2).

### Δa* (Change Along Red-Green Axis) Values

**Fig. 8:**
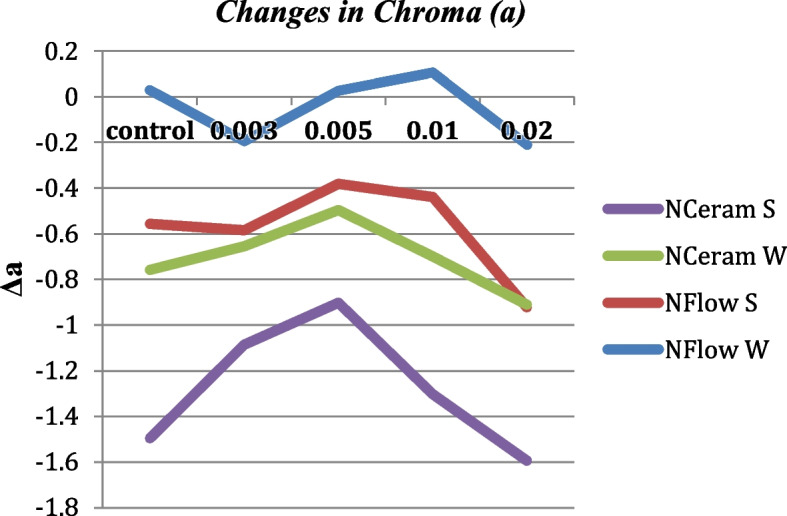
Changes in Chroma (Δa) of zMgO composites after thermocycling. NFlow W: Tetric-N-Flow wires nanoparticles; NFlow S: Tetric-N-Flow spherical nanoparticles; NCeram W: Tetric-N-Ceram wires nanoparticles; NCeram S: Tetric-N-Ceram spherical nanoparticles

**Fig. 9:**
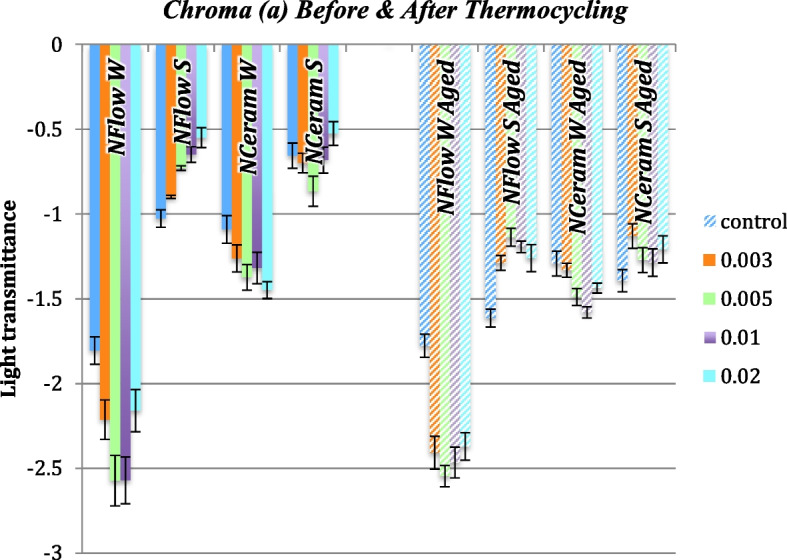
Changes in Chroma (a) of MgO nanoparticle composites before and after thermocycling. NFlow W: Tetric-N-Flow wires nanoparticles; NFlow S: Tetric-N-Flow spherical nanoparticles; NCeram W: Tetric-N-Ceram wires nanoparticles; NCeram S: Tetric-N-Ceram spherical nanoparticles

Before and after thermocycling, there was no significant variation amid the control and the groups of N-Flow and N-Ceram reinforced with different concentrations of zMgO NPs (*p* > 0.05). Δa* values were in the range of -1.5 to 1.7. Pearson coefficient showed a weak correlation of Δa in groups 1, 2, 3, and 4, respectively (r = 0.02, 0.03, 0.3 and 0.2) (Figs. 8 and 9).

### Δb* (Change Along Yellow-Blue Axis) Values

**Fig. 10:**
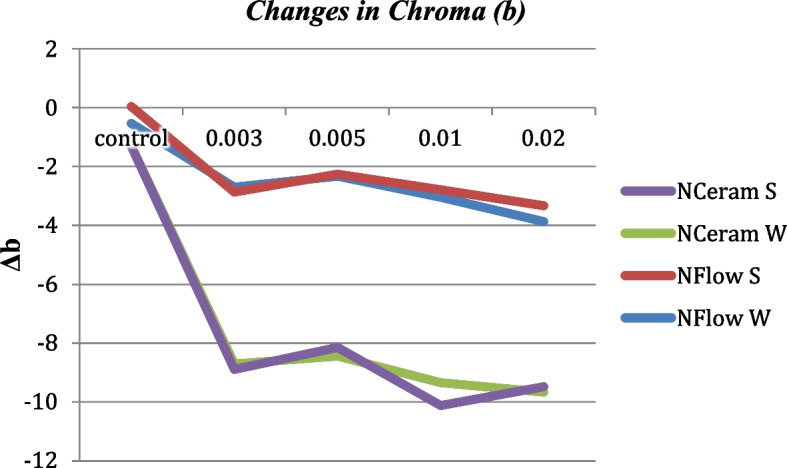
Changes in Chroma (Δb) of zMgO composites after thermocycling. NFlow W: Tetric-N-Flow wires nanoparticles; NFlow S: Tetric-N-Flow spherical nanoparticles; NCeram W: Tetric-N-Ceram wires nanoparticles; NCeram S: Tetric-N-Ceram spherical nanoparticles

**Fig. 11:**
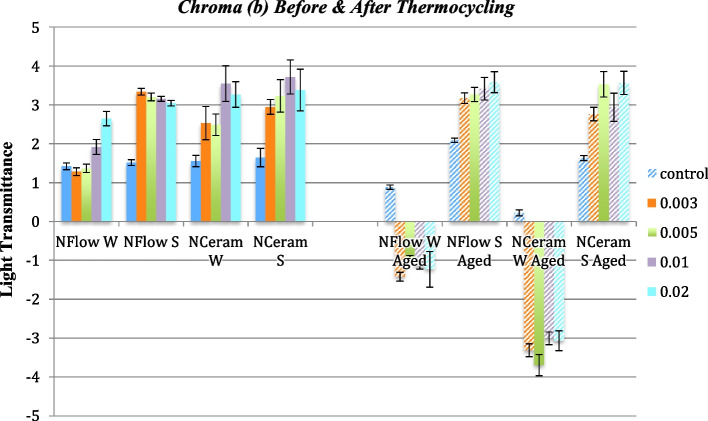
Changes in Chroma (b) of zMgO composites before and after thermocycling. NFlow W: Tetric-N-Flow wires nanoparticles; NFlow S: Tetric-N-Flow spherical nanoparticles; NCeram W: Tetric-N-Ceram wires nanoparticles; NCeram S: Tetric-N-Ceram spherical nanoparticles

Before thermocycling, there was no significant variation amid the different groups (*p* > 0.05). While after thermocycling, there was a statistically significant difference in N-Flow wires and N-Ceram wires between the control and the different groups (*p* < 0.05). The Tukey test showed no significant variation amid the groups with different zMgO concentrations (*p* > 0.05). Pearson coefficient showed a weak correlation of Δb in groups 1, 2, 3 and 4 respectively (r = 0.12, 0.1, 0.03 and 0.02) (Figs. 10 and 11).

## Discussion

In the oral cavity, dental composite restorations are continuously exposed to various discoloration agents present in food and beverages. The degree of discoloration of composite restorations is influenced by numerous factors, such as inadequate polymerization [34, 35], water absorption, [38, 39] chemical reactivity [1], diet [40], oral hygiene, [41] and surface roughness of the restoration [42].

This research is part of a big project evaluating the effect of zMgO NPs on composite from all different biological, mechanical, and physical aspects. It involves the investigation of the characterization [34], cytotoxicity [33], and antimicrobial properties [32] of zMgO NPs as well as their antimicrobial effect when added to dental composites, [43] dental cements [30], mouth washes, [31] and their effect on the mechanical properties, [43] and surface roughness [44]. Their effect on enhancing enamel remineralization was also investigated [45], along with their ability of bone remineralization in diabetics [46]. In this study, the physical aspect of the color stability was investigated by adding zein-coated MgO nanoparticles of two shapes (nanowires and nanospheres) to two types of composite restorations (N-Flow and N-Ceram).

Characterization of the new hybrid inorganic composite is crucial in order to understand the properties of the new material, as well as to gain the capacity to manipulate it with confidence. X-ray diffraction analysis (XRD), Field Emission Scanning Electron Microscopy **(**FESEM) and Fourier Transform Infrared Spectroscopy (FTIR) are effective to probe a multicomponent structure, such as composite, because it provides information for both composition and metal-polymer interactions, stipulating a fundamental understanding of inorganic-polymer interaction mechanisms. Characterization of N-Flow enhanced with zMgO with XRD and FTIR revealed the formation of a new hybrid composite that maintained the original properties of the composite (Figs. 2 and 4). Furthermore, the FESEM showed a uniform distribution of zMgO NPs in the N-Flow matrix, which helps to preserve the nano-properties of zMgO NPs (Fig. 3).

Spectrophotometry was used to avoid all bias due to the subjectivity of the human eye evaluation, in accordance with previous studies [39, 47]. This evaluation can simulate the clinical situation in which at least one of the walls of the cavity is present, that is—class I, II, and III restorations and veneers [48].

Visible color is made of red, green, and blue nominated as the three primary colors. The L*a*b* color scope is illustrated by homogenous chromaticities. L* is the color value (black to white), whereas (a*b*) is the chroma where (+ a*) is red, (− a*) is green, (+ b*) is yellow, and (− b*) is blue and the delta (Δ E) value is the change in the overall color stability. According to the National Bureau of Standards, a color change ΔE < 1 is recognized as very low. When ΔE is in the range of 1—3.3 it is clinically acceptable, and when ΔE > 3.7 it is clinically observable [49]. Results revealed that enhancing N-Flow and N-Ceram composite resins with different concentrations of antimicrobial zMgO nanowires or nanospheres did not alter the total color stability of N-Flow and N-Ceram composites. ΔE was between 1 and 2.5, which is in the accepted range (Fig. 5). The small filler size (10–60 nm) and concentration of zMgO NPs do not scatter light as it is smaller than visible light and consequently could not be noticed by the human eye. Thus, we accepted the null hypothesis that adding different shapes and concentrations of zMgO NPs preserved the color stability of the N-Ceram and N-Flow composite resin and accordingly rejected the alternate hypothesis.

Filler shape and surface modification to the resin have serious effects on composite color stability [50]. The resin matrix is more susceptible to water sorption as the filler particles can absorb water only on the surface. The amount of matrix and the quality of the bond between the filler and the resin matrix are the two factors influencing water sorption. High filler loaded composite has higher color stability than a less loaded one, therefore increasing the longevity of the composite restoration. Additionally, the incorporation of nanoparticles could greatly reduce the polymerization shrinkage of resin composites by decreasing the resin-to-filler ratio[51].

Recently organic and inorganic nanoparticles were used as fillers in dental composite resin in order to ameliorate its properties. The high surface area and the nanoscale size give nanoparticles special chemical and physical characteristics. The variant sized nanoparticles absorb visible light differently reflecting variable colors that confer their optical properties. The size of the nanoparticles determines the color and wavelength of the scattered light. Short wavelengths of blue and violet colors are scattered by small nanoparticles while long wavelengths of red and orange colors are scattered by large nanoparticles. Large particles scatter more light than small particles. Filler size and distribution are directly affecting the optical properties of composite. Small filler size assists in maintaining color stability and enhancing esthetics [52].

Since nanoparticles may affect the CIE L*a*b* parameters differently, and to have a more detailed picture of the effect of different nanoparticles’ shapes on composite, a close view on changes in each parameter can help to better understand their behavior rather than focusing on ∆E values only.

In this study, the lightness (L) values were in accordance with literature ranging from 54 to 78 [49]. There was a significant variation amid the control and the N-Flow with 2% zMgO spheres and N-Ceram with 2% zMgO spheres before and after thermocycling (Figs. 6 and 7). This could be attributed to the size, morphology, and concentration of these nanospheres. Small round nanoparticles with high concentration facilitate their agglomeration in the resin matrix, affecting their light transmittance. While light colored specimens give a positive ΔL*, dark colored ones give a negative ΔL*[11]; in this study, ΔL* values were a mix of positive and negative. Composite reinforced with nanospheres showed negative values while those reinforced with nanowires showed a mix of positive and negative values. This could be attributed to the changing optical properties of nanowires with their concentration and their orientation in the organic matrix [53, 54]. However, changes in lightness (ΔL*) values were very small indicating better color stability for the zMgO NPs composite.

Translucency is a property resulting from examining the color difference of a material with the same specific thickness in front of a white and a black background. This color difference provides a specific value in relation to the common visual perception of translucency. It is affected by the variance amid the refractive index (RI) of the filler and matrix. The higher the RI differences between the two phases, the higher the opacity of the nanocomposite. Furthermore, the more the filler scatters light, the less noticeable and measurable the color change is. The smaller the scattering center, the less scattered light in the visible range.

Thus, fillers size affects the translucency of dental composite. When the filler size is smaller than the visible light wavelength, it will neither scatter nor absorb the light. Consequently, it will not be noticed by the human eye, which appears to explain the fact that zMgO NPs composite has no significant difference over the control ones [14, 39, 47, 55].

There was no significant variation in Chroma (a) amid the control and the groups of N-Flow and N-Ceram reinforced with different concentrations of zMgO NPs (*p* > 0.05) before and after thermocycling (Figs. 8 and 9). Chroma (a) values were in accordance with the literature ranging from -0.4 to 1.7 [49]. As the size of nanoparticles plays a significant role in the color and scattering of light, the small size of zMgO NPs (10-60nm) [34], being tinier than the light wavelength, doesn’t permit scattering or absorption of light, which in turn assists in maintaining color stability and enhancing esthetics [52, 56].

A green color is denoted by a negative Δa*, and a red color connoted a positive Δa* [13]. All the composite groups with nanospheres showed a positive Δa indicating a reflection of red color while some of the composite groups reinforced with nanowires showed a negative Δa indicating a shift towards the green color. Although this shift did not affect the total color change of the composite color reinforced with nanowires (ΔE), it could be related to the unique optical properties of nanowires that change according to their size and orientation in the resin matrix [53, 54, 57, 58].

Similarly in this study, there was no significant variation in chroma (b) amid the different groups before thermocycling (*p* > 0.05), while after thermocycling, there was a statistically significant difference in N-Flow wires and N-Ceram wires amid the control and the different groups (*p* < 0.05) (Figs. 10 and 11). This could be attributed to the shape of nanoparticles. Researchers found that non-linear nanowires can change the color of light [58]. Others found a correlation between the colors of the nanowires deposited on a solid substrate and their diameters [53]. Also, studies on silicon nanowire (NW) pairs showed that they can display a wide range of structural colors by controlling their radiative coupling [54].

Therefore, changes in (b) could be related to the change in optical properties of nanowires according to their size and orientation in the resin matrix [53, 58]. A yellow color connoted a positive Δb*, while a blue color denoted a negative Δb*. Most of Δb* values were positive, denoting a shift to yellow color except N-Flow and N-Ceram reinforced with 0.5% nanospheres and N-Ceram reinforced with 0.3% Nanowires—they had negative Δb* values indicating a shift towards blue color that could be related to the small size of these nanoparticles [52].

The incorporation of inorganic fillers in the material leads to density increase with a decrease in porosity. This antagonistic relationship between density and porosity induces less light transmittance, preserving the restoration color stability [59, 60]. This phenomenon could partially explain the color stability in the current study where the small sized zMgO nanoparticles, especially the nanospheres, could fill the spaces in the resin matrix and accordingly decrease its porosity and increase its density. Hence, they maintained the color stability of N-Flow and N-Ceram composite.

The surface modification of nanoparticles is necessary to reduce the filler surface energy and therefore reduce composite hydrophilicity. Zein coating of MgO nanoparticles enhanced their dispersion within the resin and supplied a functional interface that permitted covalent bonds between the zMgO NPs and composite matrix which in turn reduced the porosity in the materials [12] and preserved the color stability of N-Flow and N-Ceram composite resins.

There are some limitations with this study, as with other in vitro investigations. The study conditions do not fully simulate the oral environment. Moreover, future in vivo and clinical studies are required to evaluate the washing effect of saliva and the effect of their enzymatic action on the color stability of composite with different nanoparticles shapes.

## Conclusion

Enhancing N-Ceram and N-Flow composite resins with different concentrations of antimicrobial zMgO nanowires and nanospheres does not alter the total color stability of these materials before and after thermocycling. Considerations can be made to incorporate zMgO into composite for clinical use to improve treatment when the benefits of the antimicrobial aspect are desired.

## Data Availability

Data is available from the corresponding author upon reasonable request.

## Abbreviations

MgO: Magnesium Oxide
MgO NPs: Magnesium Oxide nanoparticles
zMgO NPs: Zein-incorporated Magnesium Oxide nanoparticles
FESEM: Field Emission Scanning Electron Microscopy
FTIR: Fourier Transform Infrared
XRD: X-Ray Diffraction
